# (*E*)-2-Hy­droxy­cinnamaldehyde

**DOI:** 10.1107/S1600536813006648

**Published:** 2013-03-16

**Authors:** Ki-Tae Kang, Sung-Gon Kim

**Affiliations:** a Department of Chemistry, Kyonggi University, San 94-6, Iui-dong, Yeongtong-gu, Suwon 443-760, Republic of Korea

## Abstract

The asymmetric unit of the title compound, C_9_H_8_O_2_, contains two independent mol­ecules, both of which are essentially planar (r.m.s. deviations = 0.0294 and 0.0284 Å). The C=C double bond is in an *E* conformation and the vinyl­aldehyde groups adopt extended conformations. In the crystal, mol­ecules are linked by O—H⋯O hydrogen bonds, forming infinite chains parallel to [101].

## Related literature

For the synthesis of the title compound, see: Kim *et al.* (2004 ▶); Zeiter & Rose (2009 ▶). For the biological activity of 2-hy­droxy­cinnamaldehydes, see: Kwon *et al.* (1996 ▶); Lee *et al.* (1999 ▶); Ka *et al.* (2003 ▶). For applications of 2-hy­droxy­cinnamaldehydes, see: Zu *et al.* (2009 ▶); Choi & Kim (2010 ▶); Lee & Kim (2011 ▶).
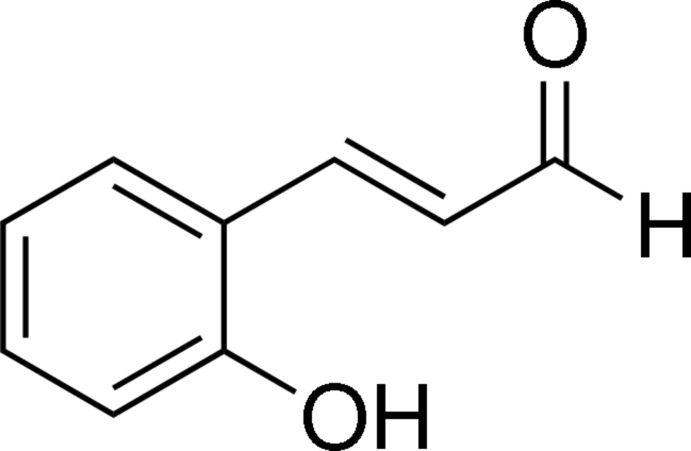


## Experimental

### 

#### Crystal data

C_9_H_8_O_2_
*M* *_r_* = 148.15
Monoclinic, 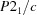

*a* = 10.1192 (15) Å
*b* = 13.7078 (19) Å
*c* = 10.9891 (15) Å
β = 102.537 (3)°
*V* = 1488.0 (4) Å^3^
*Z* = 8
Mo *K*α radiation
μ = 0.09 mm^−1^
*T* = 200 K
0.40 × 0.34 × 0.29 mm

#### Data collection

Bruker SMART APEX CCD diffractometer
10982 measured reflections
3725 independent reflections
1785 reflections with *I* > 2σ(*I*)
*R*_int_ = 0.041

#### Refinement

*R*[*F* ^2^ > 2σ(*F* ^2^)] = 0.052
*wR*(*F* ^2^) = 0.182
*S* = 0.97
3725 reflections
201 parameters
H-atom parameters constrained
Δρ_max_ = 0.22 e Å^−3^
Δρ_min_ = −0.27 e Å^−3^

### 

Data collection: *SMART* (Bruker, 2007 ▶); cell refinement: *SAINT* (Bruker, 2007 ▶); data reduction: *SAINT*; program(s) used to solve structure: *SHELXTL* (Sheldrick, 2008 ▶); program(s) used to refine structure: *SHELXTL*; molecular graphics: *ORTEP-3 for Windows* (Farrugia, 2012 ▶); software used to prepare material for publication: *SHELXTL* and *publCIF* (Westrip, 2010 ▶).

## Supplementary Material

Crystal structure: contains datablock(s) I, global. DOI: 10.1107/S1600536813006648/fy2086sup1.cif

Structure factors: contains datablock(s) I. DOI: 10.1107/S1600536813006648/fy2086Isup2.hkl

Supplementary material file. DOI: 10.1107/S1600536813006648/fy2086Isup3.cml

Additional supplementary materials:  crystallographic information; 3D view; checkCIF report

## Figures and Tables

**Table 1:** Hydrogen-bond geometry (Å, °)

*D*—H⋯*A*	*D*—H	H⋯*A*	*D*⋯*A*	*D*—H⋯*A*
O3—H3*A*⋯O2^i^	0.84	1.90	2.7260 (19)	166
O1—H1*A*⋯O4^ii^	0.84	1.90	2.7193 (19)	166

Symmetry codes: (i) 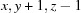; (ii) 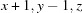.

**Fig. 1.**
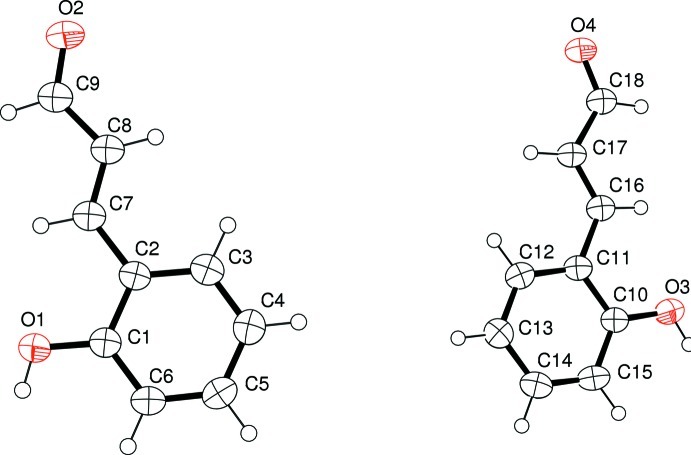
A view of the molecular structure of the title compound, showing the atom-numbering scheme. Displacement ellipsoids are drawn at the 50% probability level.

**Fig. 2.**
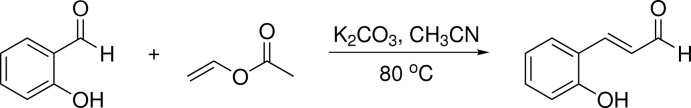
The preparation of the title compound.

## supplementary crystallographic information

### Comment

2-Hydroxycinnamaldehyde, isolated from the stern bark of *Cinnamonum cassia*, and its synthetic derivatives have been shown to inhibit on farnesyl protein transferase *in vitro*, as well as angiogenesis, and tumor cell growth (Kwon *et al.* 1996; Lee *et al.* 1999; Ka *et al.* 2003). In view of these potential applications and in continuation of our work, the structure of the title compound has been determined and the results are presented here.

X-ray analysis confirms the molecular structure and atom connectivity as illustrated in Fig. 1. The asymmetric unit of the title compound contains two independent molecules, A and B, with similar conformations. Both molecules are essentially planar. The r.m.s deviations of the atoms from their mean plane in molecules A and B are 0.0294 Å and 0.0284 Å, respectively. The molecule displays a *trans* configuration with respect to the C═C double bond, and the vinylaldehyde groups adopt extended conformations as can be seen from the torsion angles C2—C7—C8—C9 = -177.1 (2)° and C11—C16—C17—C18 = 179.2 (2)°. In the crystal, the molecules are linked by intermolecular O—H···O hydrogen bonds (Table 1).

### Experimental

A solution of 2-hydroxybenzaldehyde (10.0 mmol) and vinyl acetate (11.0 mmol) in CH_3_CN (20 ml) was added to a stirred suspension of K_2_CO_3_ in CH_3_CN (30 ml). After refluxing for 48 h, the reaction mixture was poured into cold water and diluted with EtOAc. The organic layer was washed with 10% NaOH solution, and the aqueous layer was separated, acidified with 10% HCl solution, and extracted with CH_2_Cl_2_. The resultant organic layer was dried over MgSO_4_ and concentrated *in vacuo*. The dark residue was purified by silica gel chromatography to afford the title compound (Fig. 2). Crystals suitable for X-ray analysis were obtained by slow evaporation from an n-hexane/CH_2_Cl_2_ solution.

### Refinement

All H atoms were positioned geometrically (O—H = 0.84 Å and C—H = 0.95 Å) and constrained to ride on their parent atoms, with *U*_iso_(H) = 1.2*U*_eq_(C) or *U*_iso_(H) = 1.5*U*_eq_(O). The orientations of the H atoms in the hydroxyl groups were refined using a rotating rigid group approximation.

### Crystal data

**Table:**

C_9_H_8_O_2_	*F*(000) = 624
*M**_r_* = 148.15	*D*_x_ = 1.323 Mg m^−^^3^
Monoclinic, *P*2_1_/*c*	Mo *K*α radiation, λ = 0.71073 Å
Hall symbol: -P 2ybc	Cell parameters from 3327 reflections
*a* = 10.1192 (15) Å	θ = 2.4–28.3°
*b* = 13.7078 (19) Å	µ = 0.09 mm^−^^1^
*c* = 10.9891 (15) Å	*T* = 200 K
β = 102.537 (3)°	Block, pale yellow
*V* = 1488.0 (4) Å^3^	0.40 × 0.34 × 0.29 mm
*Z* = 8	

### Data collection

**Table:**

Bruker SMART APEX CCD diffractometer	1785 reflections with *I* > 2σ(*I*)
Radiation source: fine-focus sealed tube	*R*_int_ = 0.041
Graphite monochromator	θ_max_ = 28.4°, θ_min_ = 2.1°
φ and ω scans	*h* = −13→12
10982 measured reflections	*k* = −18→16
3725 independent reflections	*l* = −14→14

### Refinement

**Table:**

Refinement on *F*^2^	Primary atom site location: structure-invariant direct methods
Least-squares matrix: full	Secondary atom site location: difference Fourier map
*R*[*F*^2^ > 2σ(*F*^2^)] = 0.052	Hydrogen site location: inferred from neighbouring sites
*wR*(*F*^2^) = 0.182	H-atom parameters constrained
*S* = 0.97	*w* = 1/[σ^2^(*F*_o_^2^) + (0.0927*P*)^2^] where *P* = (*F*_o_^2^ + 2*F*_c_^2^)/3
3725 reflections	(Δ/σ)_max_ < 0.001
201 parameters	Δρ_max_ = 0.22 e Å^−^^3^
0 restraints	Δρ_min_ = −0.27 e Å^−^^3^

### Special details

**Table:**

Geometry. All e.s.d.'s (except the e.s.d. in the dihedral angle between two l.s. planes) are estimated using the full covariance matrix. The cell e.s.d.'s are taken into account individually in the estimation of e.s.d.'s in distances, angles and torsion angles; correlations between e.s.d.'s in cell parameters are only used when they are defined by crystal symmetry. An approximate (isotropic) treatment of cell e.s.d.'s is used for estimating e.s.d.'s involving l.s. planes.
Refinement. Refinement of *F*^2^ against ALL reflections. The weighted *R*-factor *wR* and goodness of fit *S* are based on *F*^2^, conventional *R*-factors *R* are based on *F*, with *F* set to zero for negative *F*^2^. The threshold expression of *F*^2^ > σ(*F*^2^) is used only for calculating *R*-factors(gt) *etc*. and is not relevant to the choice of reflections for refinement. *R*-factors based on *F*^2^ are statistically about twice as large as those based on *F*, and *R*-factors based on ALL data will be even larger.

### Fractional atomic coordinates and isotropic or equivalent isotropic displacement parameters (Å^2^)

**Table:**

	*x*	*y*	*z*	*U*_iso_*/*U*_eq_	
C1	1.0099 (2)	0.23356 (14)	0.6613 (2)	0.0446 (5)	
O1	1.00066 (17)	0.13543 (10)	0.66051 (15)	0.0594 (5)	
H1A	1.0513	0.1121	0.6166	0.089*	
C2	0.9346 (2)	0.28553 (14)	0.73291 (19)	0.0407 (5)	
C3	0.9417 (2)	0.38675 (15)	0.7331 (2)	0.0483 (6)	
H3	0.8915	0.4230	0.7813	0.058*	
C4	1.0197 (2)	0.43568 (16)	0.6651 (2)	0.0568 (7)	
H4	1.0226	0.5049	0.6658	0.068*	
C5	1.0941 (2)	0.38336 (16)	0.5954 (2)	0.0526 (6)	
H5	1.1484	0.4169	0.5484	0.063*	
C6	1.0897 (2)	0.28362 (15)	0.5939 (2)	0.0494 (6)	
H6	1.1416	0.2483	0.5463	0.059*	
C7	0.8519 (2)	0.23186 (15)	0.80343 (19)	0.0447 (5)	
H7	0.8512	0.1629	0.7948	0.054*	
C8	0.7771 (2)	0.26827 (15)	0.8786 (2)	0.0476 (6)	
H8	0.7705	0.3369	0.8879	0.057*	
C9	0.7067 (2)	0.20404 (16)	0.94542 (19)	0.0475 (6)	
H9	0.7137	0.1361	0.9307	0.057*	
O2	0.63875 (17)	0.22802 (11)	1.01920 (14)	0.0556 (5)	
C10	0.5413 (2)	1.02655 (15)	0.18904 (19)	0.0449 (5)	
O3	0.50581 (16)	1.12074 (10)	0.16394 (15)	0.0573 (5)	
H3A	0.5548	1.1450	0.1191	0.086*	
C11	0.4817 (2)	0.97679 (15)	0.27481 (19)	0.0437 (5)	
C12	0.5162 (2)	0.87942 (15)	0.3007 (2)	0.0470 (6)	
H12	0.4756	0.8449	0.3581	0.056*	
C13	0.6071 (2)	0.83215 (16)	0.2456 (2)	0.0513 (6)	
H13	0.6300	0.7659	0.2651	0.062*	
C14	0.6654 (2)	0.88235 (16)	0.1606 (2)	0.0545 (6)	
H14	0.7278	0.8500	0.1212	0.065*	
C15	0.6336 (2)	0.97798 (16)	0.1334 (2)	0.0531 (6)	
H15	0.6750	1.0116	0.0759	0.064*	
C16	0.3859 (2)	1.02833 (15)	0.3339 (2)	0.0472 (6)	
H16	0.3724	1.0955	0.3138	0.057*	
C17	0.3155 (2)	0.99221 (15)	0.4125 (2)	0.0468 (6)	
H17	0.3238	0.9253	0.4353	0.056*	
C18	0.2269 (2)	1.05456 (15)	0.4629 (2)	0.0498 (6)	
H18	0.2201	1.1204	0.4353	0.060*	
O4	0.15958 (16)	1.03140 (10)	0.53740 (14)	0.0556 (5)	

### Atomic displacement parameters (Å^2^)

**Table:**

	*U*^11^	*U*^22^	*U*^33^	*U*^12^	*U*^13^	*U*^23^
C1	0.0489 (13)	0.0384 (11)	0.0525 (13)	0.0014 (9)	0.0243 (11)	0.0000 (9)
O1	0.0778 (13)	0.0387 (8)	0.0785 (12)	−0.0006 (7)	0.0535 (10)	−0.0030 (7)
C2	0.0439 (13)	0.0384 (11)	0.0440 (12)	0.0026 (9)	0.0189 (10)	−0.0009 (9)
C3	0.0536 (14)	0.0434 (12)	0.0535 (14)	0.0030 (10)	0.0238 (11)	−0.0019 (10)
C4	0.0662 (17)	0.0406 (12)	0.0712 (17)	−0.0037 (11)	0.0319 (14)	−0.0005 (11)
C5	0.0594 (15)	0.0488 (13)	0.0575 (14)	−0.0061 (11)	0.0298 (12)	0.0029 (11)
C6	0.0545 (15)	0.0462 (12)	0.0560 (14)	0.0022 (10)	0.0308 (12)	−0.0004 (10)
C7	0.0497 (14)	0.0414 (12)	0.0485 (13)	0.0010 (9)	0.0228 (11)	−0.0009 (9)
C8	0.0550 (15)	0.0433 (12)	0.0518 (13)	0.0000 (10)	0.0277 (12)	−0.0024 (10)
C9	0.0543 (15)	0.0467 (12)	0.0466 (13)	−0.0003 (10)	0.0223 (11)	−0.0026 (10)
O2	0.0639 (11)	0.0573 (10)	0.0565 (10)	−0.0011 (8)	0.0368 (9)	−0.0010 (7)
C10	0.0520 (14)	0.0390 (11)	0.0509 (13)	−0.0038 (9)	0.0274 (11)	−0.0050 (9)
O3	0.0728 (12)	0.0428 (9)	0.0708 (11)	0.0018 (7)	0.0470 (9)	0.0043 (7)
C11	0.0466 (13)	0.0416 (12)	0.0493 (13)	−0.0030 (9)	0.0245 (11)	−0.0044 (9)
C12	0.0517 (14)	0.0427 (12)	0.0533 (13)	−0.0029 (10)	0.0262 (11)	0.0003 (10)
C13	0.0572 (15)	0.0413 (12)	0.0614 (14)	0.0006 (10)	0.0260 (12)	−0.0028 (10)
C14	0.0565 (15)	0.0498 (13)	0.0667 (15)	0.0036 (11)	0.0343 (13)	−0.0058 (11)
C15	0.0561 (15)	0.0541 (14)	0.0594 (15)	−0.0032 (11)	0.0353 (13)	−0.0027 (11)
C16	0.0539 (15)	0.0408 (11)	0.0549 (13)	−0.0015 (10)	0.0292 (12)	−0.0018 (10)
C17	0.0531 (14)	0.0435 (12)	0.0519 (13)	−0.0001 (10)	0.0292 (11)	−0.0023 (10)
C18	0.0561 (15)	0.0458 (13)	0.0556 (14)	−0.0004 (10)	0.0299 (12)	−0.0014 (10)
O4	0.0619 (11)	0.0516 (9)	0.0663 (10)	−0.0048 (7)	0.0421 (9)	−0.0062 (8)

### Geometric parameters (Å, º)

**Table:**

C1—O1	1.348 (2)	C10—O3	1.352 (2)
C1—C6	1.390 (3)	C10—C15	1.392 (3)
C1—C2	1.403 (3)	C10—C11	1.401 (3)
O1—H1A	0.8400	O3—H3A	0.8400
C2—C3	1.389 (3)	C11—C12	1.393 (3)
C2—C7	1.457 (3)	C11—C16	1.460 (3)
C3—C4	1.374 (3)	C12—C13	1.369 (3)
C3—H3	0.9500	C12—H12	0.9500
C4—C5	1.385 (3)	C13—C14	1.390 (3)
C4—H4	0.9500	C13—H13	0.9500
C5—C6	1.368 (3)	C14—C15	1.367 (3)
C5—H5	0.9500	C14—H14	0.9500
C6—H6	0.9500	C15—H15	0.9500
C7—C8	1.333 (3)	C16—C17	1.330 (3)
C7—H7	0.9500	C16—H16	0.9500
C8—C9	1.431 (3)	C17—C18	1.434 (3)
C8—H8	0.9500	C17—H17	0.9500
C9—O2	1.216 (2)	C18—O4	1.215 (2)
C9—H9	0.9500	C18—H18	0.9500
			
O1—C1—C6	122.46 (17)	O3—C10—C15	122.74 (18)
O1—C1—C2	117.70 (17)	O3—C10—C11	117.90 (17)
C6—C1—C2	119.84 (19)	C15—C10—C11	119.4 (2)
C1—O1—H1A	109.5	C10—O3—H3A	109.5
C3—C2—C1	118.24 (18)	C12—C11—C10	118.56 (18)
C3—C2—C7	122.64 (18)	C12—C11—C16	122.27 (18)
C1—C2—C7	119.12 (18)	C10—C11—C16	119.17 (19)
C4—C3—C2	121.5 (2)	C13—C12—C11	121.74 (19)
C4—C3—H3	119.2	C13—C12—H12	119.1
C2—C3—H3	119.2	C11—C12—H12	119.1
C3—C4—C5	119.6 (2)	C12—C13—C14	119.1 (2)
C3—C4—H4	120.2	C12—C13—H13	120.4
C5—C4—H4	120.2	C14—C13—H13	120.4
C6—C5—C4	120.3 (2)	C15—C14—C13	120.5 (2)
C6—C5—H5	119.9	C15—C14—H14	119.7
C4—C5—H5	119.9	C13—C14—H14	119.7
C5—C6—C1	120.53 (19)	C14—C15—C10	120.73 (19)
C5—C6—H6	119.7	C14—C15—H15	119.6
C1—C6—H6	119.7	C10—C15—H15	119.6
C8—C7—C2	127.6 (2)	C17—C16—C11	127.6 (2)
C8—C7—H7	116.2	C17—C16—H16	116.2
C2—C7—H7	116.2	C11—C16—H16	116.2
C7—C8—C9	120.0 (2)	C16—C17—C18	119.8 (2)
C7—C8—H8	120.0	C16—C17—H17	120.1
C9—C8—H8	120.0	C18—C17—H17	120.1
O2—C9—C8	126.3 (2)	O4—C18—C17	126.4 (2)
O2—C9—H9	116.9	O4—C18—H18	116.8
C8—C9—H9	116.9	C17—C18—H18	116.8
			
O1—C1—C2—C3	−179.1 (2)	O3—C10—C11—C12	179.4 (2)
C6—C1—C2—C3	0.5 (3)	C15—C10—C11—C12	−0.6 (3)
O1—C1—C2—C7	0.7 (3)	O3—C10—C11—C16	−0.6 (3)
C6—C1—C2—C7	−179.8 (2)	C15—C10—C11—C16	179.4 (2)
C1—C2—C3—C4	0.2 (3)	C10—C11—C12—C13	0.6 (3)
C7—C2—C3—C4	−179.5 (2)	C16—C11—C12—C13	−179.4 (2)
C2—C3—C4—C5	−0.6 (4)	C11—C12—C13—C14	−0.7 (4)
C3—C4—C5—C6	0.2 (4)	C12—C13—C14—C15	0.7 (4)
C4—C5—C6—C1	0.5 (4)	C13—C14—C15—C10	−0.7 (4)
O1—C1—C6—C5	178.7 (2)	O3—C10—C15—C14	−179.3 (2)
C2—C1—C6—C5	−0.8 (3)	C11—C10—C15—C14	0.7 (4)
C3—C2—C7—C8	−2.3 (4)	C12—C11—C16—C17	−3.3 (4)
C1—C2—C7—C8	178.0 (2)	C10—C11—C16—C17	176.7 (2)
C2—C7—C8—C9	−177.1 (2)	C11—C16—C17—C18	179.2 (2)
C7—C8—C9—O2	177.7 (2)	C16—C17—C18—O4	−178.2 (2)

### Hydrogen-bond geometry (Å, º)

**Table:**

*D*—H···*A*	*D*—H	H···*A*	*D*···*A*	*D*—H···*A*
O3—H3*A*···O2^i^	0.84	1.90	2.7260 (19)	166
O1—H1*A*···O4^ii^	0.84	1.90	2.7193 (19)	166

Symmetry codes: (i) *x*, *y*+1, *z*−1; (ii) *x*+1, *y*−1, *z*.
